# Multiscale simulation of the focused electron beam induced deposition process

**DOI:** 10.1038/s41598-020-77120-z

**Published:** 2020-11-30

**Authors:** Pablo de Vera, Martina Azzolini, Gennady Sushko, Isabel Abril, Rafael Garcia-Molina, Maurizio Dapor, Ilia A. Solov’yov, Andrey V. Solov’yov

**Affiliations:** 1 MBN Research Center, Altenhöferallee 3, 60438 Frankfurt am Main, Germany; 2 European Centre for Theoretical Studies in Nuclear Physics and Related Areas (ECT*), 38123 Trento, Italy; 3 Departament de Física Aplicada, Universitat d’Alacant, 03080 Alacant, Spain; 4 Departamento de Física – Centro de Investigación en Óptica y Nanofísica (CIOyN), Universidad de Murcia, 30100 Murcia, Spain; 5 Department of Physics, Carl von Ossietzky University, Carl-von-Ossietzky Straße 9-11, 26129 Oldenburg, Germany

**Keywords:** Materials chemistry, Computational chemistry, Molecular dynamics, Condensed-matter physics, Atomistic models, Design, synthesis and processing, Atomic and molecular physics

## Abstract

Focused electron beam induced deposition (FEBID) is a powerful technique for 3D-printing of complex nanodevices. However, for resolutions below 10 nm, it struggles to control size, morphology and composition of the structures, due to a lack of molecular-level understanding of the underlying irradiation-driven chemistry (IDC). Computational modeling is a tool to comprehend and further optimize FEBID-related technologies. Here we utilize a novel multiscale methodology which couples Monte Carlo simulations for radiation transport with irradiation-driven molecular dynamics for simulating IDC with atomistic resolution. Through an in depth analysis of $\hbox {W(CO)}_6$ deposition on $\hbox {SiO}_2$ and its subsequent irradiation with electrons, we provide a comprehensive description of the FEBID process and its intrinsic operation. Our analysis reveals that simulations deliver unprecedented results in modeling the FEBID process, demonstrating an excellent agreement with available experimental data of the simulated nanomaterial composition, microstructure and growth rate as a function of the primary beam parameters. The generality of the methodology provides a powerful tool to study versatile problems where IDC and multiscale phenomena play an essential role.

## Introduction

Interaction of photon, neutron and charged particle beams with matter finds plenty of technological applications, particularly in materials science and nanotechnology^1–4^. Improvements in beam focusing and control are yielding cutting-edge methodologies for the fabrication of nanometer-size devices featuring unique electronic, magnetic, superconducting, mechanical and optical properties^2, 3, 5–9^. Among them, focused electron beam induced deposition (FEBID) is especially promising, as it enables reliable direct-write fabrication of complex, free-standing 3D nano-architectures^3, 10^. Still, as the intended resolution falls below 10 nm, even FEBID struggles to yield the desired size, shape and chemical composition^10–13^, which primarily originates from the lack of molecular-level understanding of the irradiation-driven chemistry (IDC) underlying nanostructure formation and growth^10, 14^. Further progress requires to learn how to finely control IDC, a goal which will require important experimental and theoretical efforts. Multiscale simulations^15–17^ can become a powerful tool to help in this endeavour, provided that a model sufficiently accurate can be developed. This investigation aims to explore this possibility.

FEBID operates through successive cycles of organometallic precursor molecules replenishment on a substrate and irradiation by a tightly-focused electron beam, which induces the release of organic ligands and the growth of metal-enriched nanodeposits. It involves a complex interplay of phenomena, each of them requiring dedicated computational approaches: (a) deposition, diffusion and desorption of precursor molecules on the substrate; (b) multiple scattering of the primary electrons (PE) through the substrate, with a fraction of them being reflected (backscattered electrons, BSE) and the generation of additional secondary electrons (SE) by ionization; (c) electron-induced dissociation of the deposited molecules; and (d) the subsequent chemical reactions, along with potential thermo-mechanical effects^18^. While processes (b) and (c) typically happen on the femtosecond-picosecond timescale, (a) and (d) may require up to microseconds or even longer. Monte Carlo (MC) simulations have become a tool of choice for studying electron transport in condensed matter, and can also account for diffusion-reaction of molecules^19–23^, although without offering atomistic details. At the atomic/molecular level, *ab initio* methods permit the precise simulation of electronic transitions or chemical bond reorganization^24, 25^, although their applicability is typically limited to the femtosecond–picosecond timescales and to relatively small molecular sizes. In between these approaches, classical molecular dynamics (MD)^17^ and particularly reactive MD^26^ have proved to be very useful in the atomistic-scale analysis of molecular fragmentation and chemical reactions up to nanoseconds and microseconds^26, 27^. Still, a comprehensive and predictive multiscale simulation including all the FEBID-related processes has been, up to now, an elusive task.

A breakthrough into the atomistic description of FEBID was recently achieved^16^ by means of the new method that permitted simulations of irradiation-driven MD (IDMD) with the use of the software packages MBN Explorer^28^ and MBN Studio^29^. IDMD superimposes probabilities of various quantum processes (e.g., ionization, dissociative electron attachment) occurring in large and complex irradiated systems, stochastically introducing chemically reactive sites in the course of affordable reactive MD simulations. In the present investigation we utilize a combination of the aforementioned MC and IDMD methodologies and perform the first inclusive simulation of radiation transport and effects in a complex system where all the FEBID-related processes (deposition, irradiation, replenishment) are accounted for. Here specifically, detailed space-energy distributions of electrons, obtained from MC^23, 30, 31^ at different irradiation conditions, were used as an input for IDMD simulations^16, 17^ on experimentally-relevant timescales, where a direct comparison could be performed.

The coupled MC-IDMD approach was employed, for the first time, to analyze IDC at the atomistic level of detail for $\hbox {W(CO)}_6$ molecules deposited on hydroxylated $\hbox {SiO}_2$. In particular, the dependence on the primary beam energy and current of the surface morphology, composition and growth rate of the created nanostructures was analyzed and was shown to be in an excellent agreement with results of available experiments^32^. This new methodology provides the necessary molecular-level insights into the key processes behind FEBID for its further development. Furthermore, the approach being general and readily applicable to any combination of radiation type and material, opens unprecedented possibilities in the simulation of many other problems where IDC and multiscale phenomena play an essential role, including astrochemistry^33, 34^, nuclear and plasma physics^15^, radiotherapy^35, 36^ or photoelectrochemistry^37^.

## Results and discussion

Here we consider a multimolecular system, consisting of 1–2 layers of $\hbox {W(CO)}_6$ molecules deposited on a $20\times 20\,\hbox {nm}^2$ hydroxylated $\hbox {SiO}_2$ surface $(\hbox {W(CO)}_6 @\hbox {SiO}_2)$, irradiated with PE beams with a radius of $R = 5$ nm and energies $T_0=$ 0.5 – 30 keV. This specific system is commonly used in FEBID and has been extensively studied experimentally^12, 32, 38^ and theoretically^16, 24, 27^. However, it has still been impossible to reach an adequate understanding of the process, such that to provide full control of the emerging nanostructures.

The electron transport in the substrate is treated by means of the MC program SEED^30, 31^, which uses accurate inelastic^39–41^ and elastic^42^ cross sections for the interaction of electrons with condensed-phase materials as input parameters. Its coupling to MBN Explorer^28^ is done by providing energy- and space-dependent electron distributions, which determine the space-dependent rates for dissociation of molecules at the substrate surface. The interaction of the precursor molecules both with the substrate and with PE, BSE and SE is described by the IDMD method^16^. See “Methods” for further details.

In the next subsections, all stages involved in the FEBID process of $\hbox {W(CO)}_6 @ \hbox {SiO}_2$ are individually studied and the parameters affecting the simulation of the whole process are determined. Once this is done, a detailed analysis of the nanostructure growth rate, composition and microstructure as a function of the PE beam energy and current is performed.

### Precursor molecule interaction with the substrate

The first factor affecting the nanostructure growth process is the ability of the precursor molecules to migrate to the irradiated area^21^. The surface diffusion coefficient depends on the strength of the binding of the molecule to the surface, and could be determined experimentally^10, 38^. However, this is not an easy task for an arbitrary combination of precursor-substrate and temperature. Alternatively, molecular surface diffusion can be predicted by MD^16^ if the parameters for molecule-substrate interaction are known. Here, we have simulated the diffusion of $\hbox {W(CO)}_6 @ \hbox {SiO}_2$ using the MBN Explorer software^28^ by means of the procedure described earlier^16^. The obtained value of the diffusion coefficient at room temperature turned out to be $7.71\, \mu \hbox {m}^2/\hbox {s}$, being close to the experimentally determined value of $6.4 \, \mu \hbox {m} ^2/\hbox {s}$^38^. See Supplementary Information S1 for further details.

### Electron beam interaction with the substrate

The FEBID process is greatly influenced by the interaction between the PE beam and the substrate. PEs (of energies $T_0 =$ 0.5 – 30 keV in the present investigation) collide with precursor molecules, but also their multiple elastic and inelastic scattering in the substrate leads to the reflection of some of them (BSE), which re-emerge still keeping a significant fraction of their initial energy, as well as to the ionization of the medium and the production of a large number of SE with energies *T* mainly in the 1–100 eV range. PE, BSE and SE can interact with precursor molecules in very different ways, influencing the collision induced chemistry^12^, so it is essential to determine their yields and space and energy distributions.

**Figure 1:**
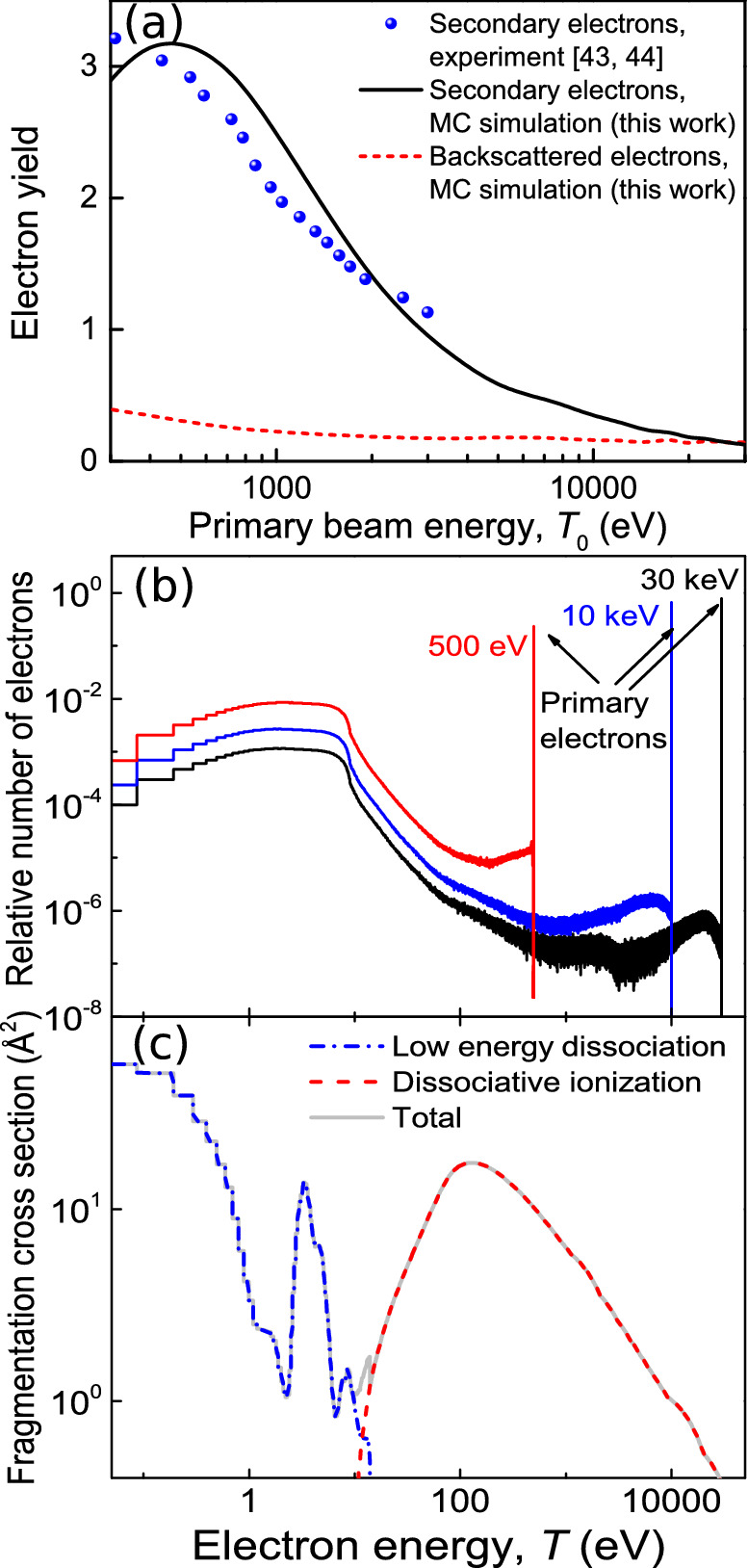
Characteristics of the electron beam and its interaction with precursor molecules. (**a**) SE and BSE yields from $\hbox {SiO}_2$ as a function of PE energy. Symbols represent experimental data^43, 44^, while lines are the results from MC simulations. The solid line shows the SE yield while the dashed line represents the BSE yield. (**b**) Energy distributions of SE and BSE crossing the $\hbox {SiO}_2$ surface for 500 eV, 10 keV and 30 keV PE. (**c**) Estimated electron-impact $\hbox {W(CO)}_6$ fragmentation cross section (solid line), with DI (dashed line) and low energy fragmentation (dash-dotted line) contributions.

MC simulations allow the analysis of the BSE and SE yields (total number of BSE and SE ejected per PE) as a function of the beam energy $T_0$. The SE yield is available experimentally for $\hbox {SiO}_2$^43, 44^ and is shown by symbols in Figure 1(a) together with the present simulation results (solid line), which reproduce the main experimental features. The BSE yield (dashed line) is rather small, although comparable to the SE yield at large energies $(T_0\simeq 20-30\, \hbox {keV})$.

Figure 1(b) shows the relative number of electrons reaching the $\hbox {SiO}_2$ surface with different energies. It can be seen that, for all PE energies $T_0$, there is an intense SE peak at low energies, with its maximum at $T < 10$ eV, while the number of BSE (those with larger energies closer to $T_0$) is in general small. Further benchmarks of energy distributions against experimental data appear in Supplementary Information S2.

**Figure 2:**
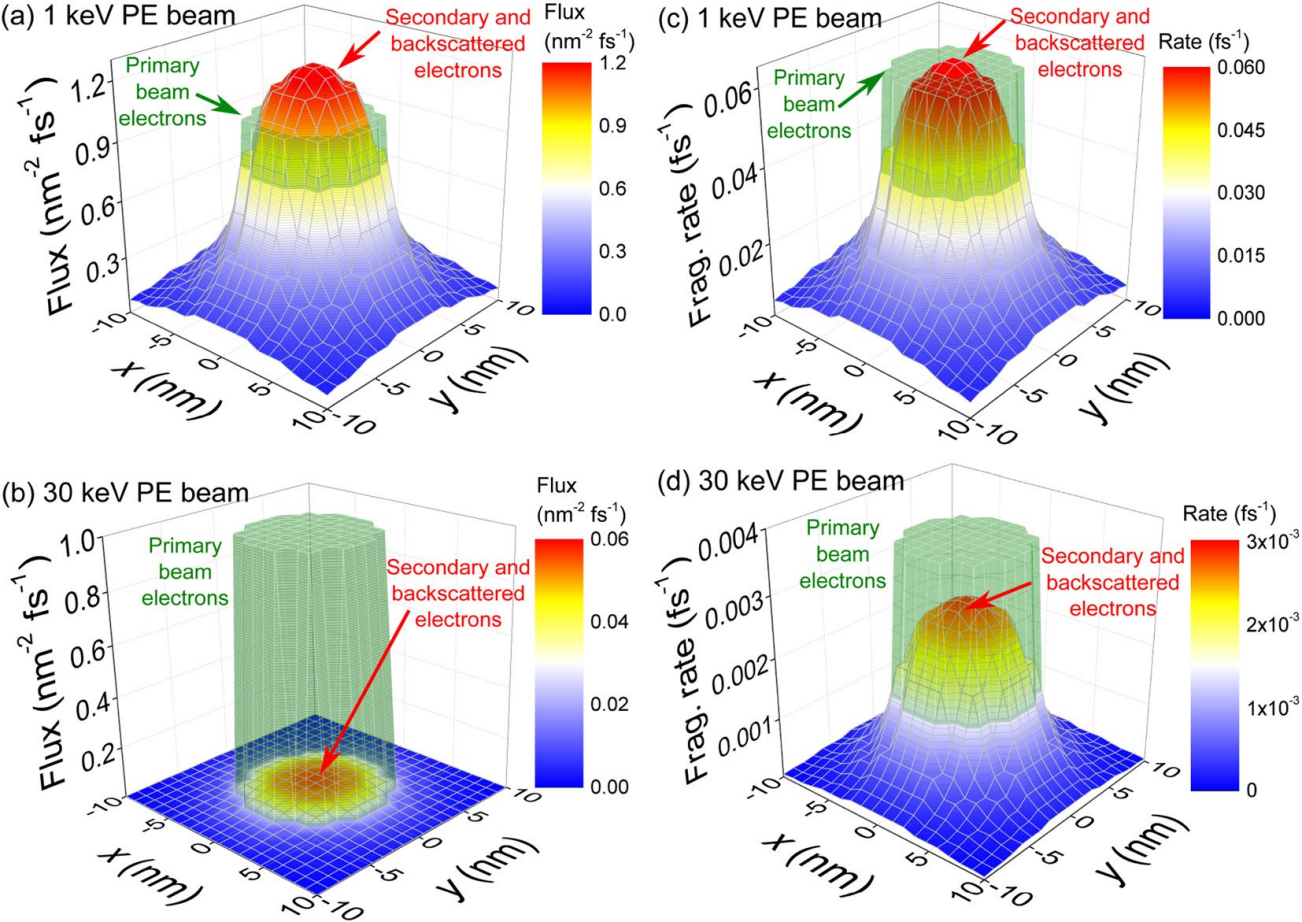
Electron fluxes and induced molecular fragmentation probabilities on the substrate surface. (**a**,**b**) Space-dependent electron fluxes on a $\hbox {SiO}_2$ surface irradiated with a uniform PE beam of 5 nm radius, flux $J_0 = 1\, \hbox {nm}^{-2}\, \hbox {fs}^{-1}$ and energy of (**a**) 1 keV and (**b**) 30 keV. The green transparent surface depicts the PE flux in the beam area, while the colored surface shows that due to SE and BSE. (**c**,**d**) $\hbox {W(CO)}_6$ fragmentation rate for (**c**) 1 keV and (**d**) 30 keV PE beams.

MC simulations also provide the space- and energy-dependent fluxes *J*(*x*, *y*, *T*) (electrons per unit area and unit time) of BSE and SE crossing the $\hbox {SiO}_2$ surface at different positions. These are shown in Figures 2(a) and (b) for uniform PE beams of 1 keV and 30 keV, respectively, and unit PE fluxes $J_0 = 1 \,\hbox {nm}^{-2}\,\hbox {fs}^{-1}$ within a circular area of radius $R=5\,\hbox {nm}$. While the high energy 30 keV beam produces a small number of SE and BSE everywhere, the lower energy 1 keV beam produces a large number of SE and BSE, which spread outside the area covered by the PE beam and exceed the number of PE at the center of the beam.

### Electron-impact molecular fragmentation cross sections

Not only the number of electrons influences the properties of the structures emerging on the surface, but also the energy-dependent probability for $\hbox {W(CO)}_6$ molecule fragmentation, given by the corresponding cross section $\sigma _{\mathrm{frag}}(T)$, has an impact. This cross section includes dissociative ionization (DI) for energies above the ionization threshold ($\sim 8.5$ eV^45^) as well as dissociative electronic excitations and dissociative electron attachment^12^.

Measurement of $\sigma _{\mathrm{frag}}(T)$ for the molecular fragmentation channels on the substrate is rather complicated, since the influence of all PE, BSE and SE crossing the surface cannot be disentangled. Under these conditions, what is usually measured is an effective decomposition cross section due to a PE beam of energy $T_0$, $\sigma _{\mathrm{decomp}}(T_0)$. Alternatively, gas-phase data may be used as a first approximation for the actual cross section $\sigma _{\mathrm{frag}}(T)$. For $\hbox {W(CO)}_6$ molecules, experimental information is available for DI^45^ and lower energy dissociation channels^46^ *relative* cross sections, but not the *absolute* values needed for the simulations. The absolute DI cross section can be calculated by means of the dielectric formalism^41^. The corresponding result is shown in Figure 1(c) by a dashed line. For energies below 14 eV, the experimental relative cross sections^46^ can be scaled in order to get a decomposition cross section $\sigma _{\mathrm{decomp}}(T_0)$ for 30 keV electrons incident in $\hbox {W(CO)}_6$@$\hbox {SiO}_2$ coinciding with the experimentally reported value^47^ (see Supplementary Information S3 for the details of the scaling procedure). The resulting low energy and total fragmentation cross sections appear in Figure 1(c) as dash-dotted and solid lines, respectively. DI dominates above $\sim$ 12 eV, while a large fraction of SE will fragment precursor molecules through the lower energy dissociation channels.

### Simulation of the FEBID process

The FEBID process relies on successive cycles of electron irradiation and precursor molecule replenishment^3, 10^. The irradiation phases are simulated by means of the IDMD method^16^ by evaluating space-dependent bond dissociation rates for molecules on the substrate, which are calculated as explained in “Methods”. In brief, these rates depend, in steady-state conditions, on (i) the number and energies of the electrons crossing the $\hbox {SiO}_2$ surface at each point per unit time and unit area (which in turn are determined by the PE beam energy $T_0$ and flux $J_0$), and (ii) the energy-dependent molecular fragmentation cross section $\sigma _{\mathrm{frag}}(T)$.

Figures 2(c) and (d) illustrate the space-dependent fragmentation rates induced by uniform 1 keV and 30 keV beams, respectively, of unit PE flux $J_0 = 1\,\hbox {nm}^{-2}\,\hbox {fs}^{-1}$ within a circular area of radius $R=5\,\hbox {nm}$. Although the number of BSE/SE electrons for 30 keV is small, their large cross section (in relation to PE) produces a significant fragmentation probability, but less than that due to PE at the beam area. However, for 1 keV, the fragmentation probability due to BSE/SE ($\sim$ 80–90 % exclusively due to SE) is very large, and significantly extends beyond the PE beam area. These results clearly demonstrate the very different scenarios to be expected for beams of different energies and which will importantly influence the deposit properties, as well as the prominent role of low-energy SE on molecular fragmentation. It is important to note that both the number and energies of electrons and their energy-dependent molecular fragmentation cross sections influence the growth mechanisms, which can be differently influenced by PE and BSE/SE electrons for different PE beam energies, precursor molecules and substrates^48^.

**Figure 3:**
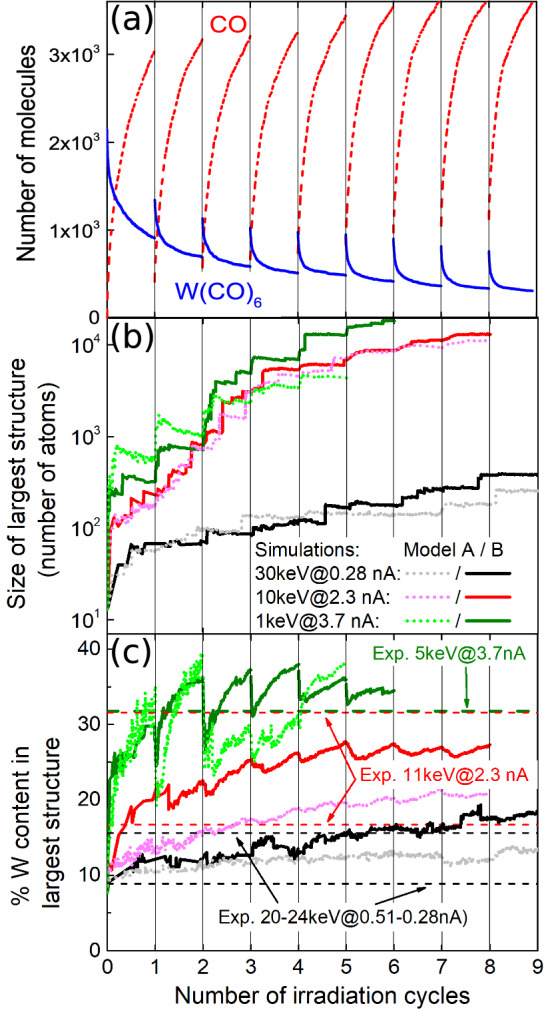
Evolution of the chemical species on the substrate surface during several FEBID cycles. (**a**) Time evolution of the number of $\hbox {W(CO)}_6$ molecules on $\hbox {SiO}_2$ (solid line) and free CO molecules (dashed line) during FEBID with a 30keV@5.9 nA beam. (**b**) Evolution of the number of atoms and (**c**) the W-metal content in the largest simulated islands for PE beams of energies 1, 10 and 30 keV, for different currents. Dotted and solid curves depict the results from two different chemistry models, in which dangling bonds within the same growing nanostructure are (model B) or are not (model A) allowed to recombine. Dashed horizontal lines, pointed by arrows, correspond to experimentally obtained compositions at the conditions indicated by the corresponding label^32^.

Each irradiation phase lasts for a time known as dwell time, whose typical duration in experiment $(\ge \mu \,\hbox {s})$ is still computationally demanding for MD. Instead, they are set here to 10 ns. Consequently, simulated PE fluxes $J_0$ (and hence PE beam currents $I_0$) must be scaled to match the same number of PE per unit area and per dwell time as in experiments^16^ (see Supplementary Information S4.A). As for replenishment, its characteristic times are also typically very long $(\sim \,\hbox {ms})$. In simulations, the CO molecules desorbed to the gas phase are simply removed during the replenishment stages and new $\hbox {W(CO)}_6$ molecules are deposited. Figure 3(a) illustrates these successive irradiation-replenishment stages by depicting the number of $\hbox {W(CO)}_6$ and free CO molecules during several of these cycles for a 30 keV PE beam of equivalent experimental current $I_0^{\exp} = 5.9\, \hbox {nA}$ (in short, 30keV@5.9nA).

As the irradiation-replenishment cycles proceed, the process of nucleation of metal-enriched islands and its coalescence starts^16^. This is shown in Figure 3(b), where the number of atoms (either W, C or O) in the largest island is shown for three simulation conditions close to reported in experiments^32^: 30keV@0.28nA, 10keV@2.3nA and 1keV@3.7nA. During the irradiation-replenishment cycles, a number of islands or atomic nanoclusters of different sizes and compositions appear on the substrate as the result of IDC. For the sake of clarity, only the size of the largest of these clusters is shown in Fig. 3(b). Smaller clusters tend to merge with time giving rise to larger structures and, eventually, to the largest island displayed in the figure. The jumps in the island size observed with some frequency are due to the merging of independent nanoclusters that grow on the substrate. Results of two different models for the chemistry occurring within the growing nanostructure are presented^16^: in model A (dotted lines), dangling bonds of a given nanostructure can only react with unsaturated bonds belonging to a different molecule; in model B (solid lines), the restructuring of bonds within a growing nanostructure is also allowed (see Supplementary Information S4.C for further details).

As the nanostructures grow, their average chemical composition also changes. The time evolution of the W-metal content of the largest nanoisland, for the three aforementioned combinations of PE beam parameters, is depicted in Figure 3(c) for the chemistry models A (dotted lines) and B (solid lines). The metal content grows fast during the first irradiation cycles, until it slowly starts to saturate for each set of beam parameters after $\sim 4-5$ irradiation cycles. It is worth noting that our simulation results are consistent with experimental data^32^ for the 20–24keV@0.28–0.51nA, 11keV@2.3nA and 5keV@3.7nA cases, represented by dashed horizontal lines in Figure 3(c). It should be remarked that, as the FEBID process proceeds and the largest atomic island grows on the substrate by incorporating smaller clusters, the atomic content of the former evolves towards the average composition of the entire fabricated deposit, which is what can be experimentally determined.

**Figure 4:**
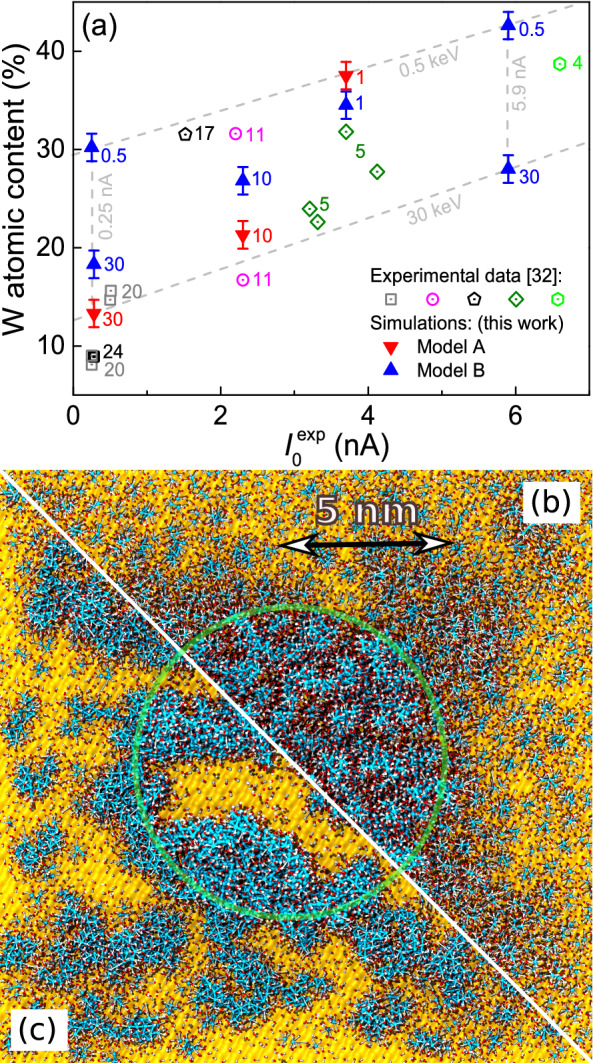
Compositions and morphologies of the deposits created by FEBID. (**a**) Dependence of the deposit metal content on the beam energy $T_0$ and current $I_0^{\exp}$, from experiments (open symbols)^32^ and simulations (full symbols). Numbers next to symbols represent the beam energy in keV for each case. Lower panels show the top views of the deposits produced by (**b**) 10keV@2.3nA and (**c**) 1keV@3.7nA beams. The green area marks the PE beam spot while blue, white and red spheres represent, respectively, W, C and O atoms; the $\hbox {SiO}_2$ substrate is represented by a yellow surface.

Experimental measurements were limited to particular values of energy and current due to the characteristics of the electron source^32^. Nonetheless, our simulation method allows for the exploration of much wider regions of electron beam parameters. To do so, we also considered the cases of 30keV@5.9nA, 0.5keV@0.25nA and 0.5keV@5.9nA, obtaining the deposit metal contents depicted by full symbols in Figure 4(a), as a function of experimentally equivalent current $I_0^{\exp}$. Error bars show the standard deviations obtained from three independent simulations for each case. Experimental results^32^ are shown by open symbols. Numbers next to symbols represent the beam energies in keV. It is clearly seen that the results from simulations are within the range of experimental uncertainties, which indicates the predictive capabilities of the simulations.

The cases analyzed in this investigation provide a detailed “map” of the attainable metal contents in the deposits as a function of the beam parameters, which is a very valuable outcome for the optimization of FEBID with $\hbox {W(CO)}_6 @ \hbox {SiO}_2$. This is marked in Figure 4(a) by dashed lines corresponding to the limiting values of energy and current studied. These results clearly show that, within the analyzed energy domain, a decrease in the beam energy and an increase in the current promote the faster growth of the deposit, as well as the augment in its metal content. Simulation results provide the grounds for clearly understanding such trends: an increment in the current means a larger number of PE per unit time, while a reduction in the energy produces an increase in the SE yield (Figure 1(a)). These lead to both the greater size of the deposit and its larger metal content due to the increased probability for bond cleavage (Figures 2(c)-(d)). It should be noted that a reduction of beam energy below $\sim 400\,\hbox {eV}$ may diminish the metal content due to the lowering of the electron yields (Figure 1).

Finally, Figures 4(b) and (c) show top views of the simulated deposits for 1keV@3.7nA and 10keV@2.3nA, after 5 and 7 irradiation cycles, respectively (the number of atoms in the largest island is similar in these cases, $\sim 12000$). The green circular line marks the area covered by the PE beam (having a radius of 5 nm). These figures help to understand how different energy-current regimes can lead to distinct deposit microstructures and edge broadenings. While the higher energy beam of 10 keV produces a deposit almost entirely localized within the intended nanomanufacturing region (i.e., the PE beam area), the lower energy beam of 1 keV produces a more sparse and ramified deposit (at least during the early stage of the FEBID process), that significantly extends beyond the PE beam area, producing an undesired edge broadening of the structure. In the present investigation we focus on better understanding the initial stages of the FEBID process (just a few irradiation cycles), which can be currently monitored in experiments with electron microscopy^49^. Such experiments should bring (together with these simulations) additional atomistic insights into FEBID, and would also serve for checking the quality of the MC-IDMD modeling. The anisotropy observed in the 1 keV case is due to this reduced number of irradiation cycles performed in this investigation. Although a detailed analysis of these effects deserves a more in-depth analysis (which is not possible within the limits of the present manuscript and would require additional computational efforts), it is worth to note that the SE yield goes from larger than 1 to lower than 1 in the 1–10 keV range (Figure 1(a)), SE being the main responsible for the beam halo (Figure 2). The effect of PE beam energy on the edge broadening of the manufactured deposit was previously analyzed by means of MC simulations^50, 51^, which indicate similar trends as discussed here. However, it should be noted that the present methodology allows studying not only the space distribution of the deposit, but also its microstructure and composition with atomistic resolution. Such detailed predictions on the early stage of growth of metal deposits can be currently tested experimentally^49^.

## Conclusions

In this study we have demonstrated how to couple detailed space and energy distributions of electrons at the substrate surface (obtained from MC calculations^23, 30, 31^) with radiation-induced dynamics and chemical reactions simulations (by means of the IDMD technique^16, 17^) in order to describe radiation effects at the molecular level for experimentally relevant timescales. As a particular case study, and due to its relevance in nanotechnology, we have analyzed the FEBID process for $\hbox {W(CO)}_6$ precursor molecules on hydroxylated $\hbox {SiO}_2$.

The presented results demonstrate how the novel MC-IDMD approach provides the necessary molecular insights into the key processes behind FEBID, which can be used for its further optimization and development. Notably, the simulations (which rely on basic atomic and molecular data such as cross sections for electron scattering and molecular fragmentation) have demonstrated a great predictive power, yielding, for the first time, fabricated nanostructure compositions and morphologies in excellent agreement with available experimental data^32^. Particularly, the increase in both the growth rate and W-metal content of the deposits with the increase in PE beam current and with the decrease in its energy, have been shown to be related to the increase in the number of ejected low energy SE. The latter are also responsible for the different microsrtuctures and edge broadenings observed for beams of different energies. Many other aspects influencing FEBID and not addressed here (namely, other substrate-molecule combinations, different replenishment conditions^38^, the effects of contaminants or local heating by the PE beam^18^, post-growth purification procedures...) can be analyzed by utilizing the protocols described in the present investigation.

Moreover, the new introduced methodology, which bridges the gap between other current approaches to describe radiation-induced effects spanning multiple space, time and energy scales, is general and readily applicable in many other important fields. It is worth noticing that mechanisms rather similar to the ones underlying FEBID (i.e., electron generation by different types of radiation, their transport and the chemistry induced on surfaces) are common to problems as diverse as the astrochemistry processes happening in interstellar ices due to cosmic radiation^33, 34^, in the use of metallic nanoparticles as enhancers of modern radiotherapies^35, 52^ or in photoelectrochemical devices^37^. This new MC-IDMD approach offers a valuable tool which might provide unprecedented insights in many relevant problems in physics, chemistry, materials science, biomedicine and related technologies, in which irradiation-driven chemistry and multiscale phenomena play an essential role.

## Methods

Simulations were performed by means of the irradiation driven MD (IDMD) method^16^ implemented in the MBN (Meso-Bio-Nano) Explorer software package^17, 28^. Within this framework, the space-dependent rate for bond cleavage in molecules on the substrate surface is given by:

1 $$\begin{aligned} P(x,y) = \sigma _{{\mathrm{frag}}}\left( T_0\right) \, J_{\mathrm{PE}}\left( x,y,T_0\right) + \sum _i \sigma _{{\mathrm{frag}}}\left( T_i\right) \, J_{\mathrm{SE/BSE}}\left( x,y,T_i\right) \, \text{, } \end{aligned}$$

where a discrete set of values for the electron energies $T_i$ was assumed for simplicity, but without affecting the final results. $J_{\mathrm{PE/SE/BSE}}(x,y,T_i)$ are space- and energy-dependent fluxes of PE/SE/BSE (electrons per unit area and unit time) and $\sigma _{\mathrm{frag}}(T_i)$ is the energy-dependent molecular fragmentation cross section. The PE beam flux at the irradiated circular spot of radius *R* is:

2 $$\begin{aligned} J_0 = \frac{I_0}{eS_0} \, \text{, } \end{aligned}$$

where $I_0$ corresponds to the PE beam current, $S_0 = \pi R^2$ to its area and *e* is the elementary charge. Note that $\sum _i J_{\mathrm{SE/BSE}}(x,y,T_i) = J_{\mathrm{SE/BSE}}(x,y)$ gives the space-dependent fluxes which are plotted in Figures 2(a)-(b). For uniform PE beams, as used in this investigation, $J_{\mathrm{PE}}(x,y,T_0) = J_0$ for every point with coordinates $x^2+y^2 \le R^2$.

The electron distributions were simulated using the MC radiation transport code SEED (Secondary Electron Energy Deposition)^23, 30, 31^. Molecular fragmentation and further chemical reactions were simulated by means of MBN Explorer^16, 17, 28^. Its dedicated user interface and multi-task toolkit, MBN Studio^29^, was employed for constructing the molecular system, performing the precursor molecule replenishment phases, as well as for analyzing the IDMD simulation results.

### Monte Carlo code SEED

The SEED code follows the classical trajectories of energetic electrons traveling inside a condensed phase material, by employing the usual Monte Carlo recipes for electron transport simulation^23, 30, 31^. It is based on the calculation of (i) the differential inelastic scattering cross sections accurately obtained by using the dielectric formalism^30, 39, 40^, (ii) the electron-phonon quasi-elastic scattering cross-section computed by the use of the Fröhlich theory^53^ and (iii) the differential elastic scattering cross section performed by the relativistic partial wave expansion method (RPWEM)^42^ including the Ganachaud and Mokrani empirical correction for low electron energies ($\le$ 20–30 eV)^54^.

The empirical parameters for the Fröhlich and the Ganachaud-Mokrani theories are set in order to reproduce by simulation the experimentally known SE yield for $\hbox {SiO}_2$^43, 44^. See Supplementary Information S2 and Refs.^23, 30, 31^ for extended discussions on the SEED code and its validation.

### MBN Explorer and Irradiation Driven Molecular Dynamics

MBN Explorer is a multi-purpose software package for advanced multiscale simulations of structure and dynamics of complex molecular systems^17, 28^, featuring a wide variety of computational algorithms for the simulation of atomistic and coarse-grained systems. It includes the advanced algorithms of reactive MD^26^ and the unique IDMD^16^ exploited in this investigation.

In the MD approach, the dynamics of a system is followed by numerically solving the coupled classical Langevin equations of motion of all its constituent atoms. The interaction forces are treated in this work by means of the CHARMM force field^55^.

The IDMD algorithm implemented in MBN Explorer^16^ superimposes random processes of molecular bond breakage due to irradiation during classical reactive MD. These processes are treated as local (involving the atoms participating in a chemical bond) energy deposition events occurring in the sub-femtosecond timescale, so they are considered to happen instantaneously between successive simulation time steps. They occur randomly, with a rate determined by the probabilities for quantum processes such as dissociative ionization or dissociative electron attachment, Equation (1). The fast relaxation of the excess energy after these interactions results in the cleavage of particular bonds and the formation of active species (radicals with unsaturated dangling bonds) which can undergo further chemical reactions.

The cleavage and formation of chemical bonds and the monitoring of the system’s dynamical topology, along with the redistribution of atomic partial charges, is managed by means of the reactive version of the CHARMM force field implemented in MBN Explorer^26^. Its parameterization for the $\hbox {W(CO)}_6$ molecule was described in an earlier study^27^. In this investigation we assume that every fragmentation event leads to the cleavage of a single W-C bond, while the much stronger C-O bonds will not react^27^. The energy deposited in the cleaved W-C bonds is chosen in accordance with average values obtained from mass spectrometry experiments^46, 56^ and dedicated simulations of the molecule fragmentation^27^, see Supplementary Information S4.B.

The features of the IDMD methodology are explained in Ref.^16^, and all necessary details for its application to the system studied in this investigation are given in Supplementary Information S4.

## Supplementary information

Supplementary Information 1.

## Data Availability

The datasets generated during the current study are available from the corresponding author on reasonable request.
