# Supplementary material for: Multiscale simulation of the focused electron beam induced deposition process
Source: Sci Rep. 2020 Nov 30;10:20827. doi: 10.1038/s41598-020-77120-z (PMC7705715; doi:10.1038/s41598-020-77120-z)
Supplement: Supplementary file 1 — Supplementary Information 1. [file 41598_2020_77120_MOESM1_ESM.pdf]

# Multiscale simulation of the focused electron beam induced deposition process (Supplementary Information)

Pablo de Vera[1,2,*] Martina Azzolini[3], Gennady Sushko[1], Isabel Abril[4], Rafael Garcia-Molina[2], Maurizio Dapor[3], Ilia A. Solov'yov[5,†] and Andrey V. Solov'yov[1†]

[1]*MBN Research Center, Altenhöferallee 3, 60438 Frankfurt am Main, Germany*

[2]*Departamento de Física – Centro de Investigación en Óptica y Nanofísica (CIOyN), Universidad de Murcia, E-30100 Murcia, Spain*

[3]*European Centre for Theoretical Studies in Nuclear Physics and Related Areas (ECT*), I-38123 Trento, Italy*

[4]*Departament de Física Aplicada, Universitat d'Alacant, E-03080 Alacant, Spain and*

[5]*Department of Physics, Carl von Ossietzky University, Carl-von-Ossietzky Straße 9-11, 26129 Oldenburg, Germany*

[*] pablo.vera@um.es

[†] On leave from A. F. Ioffe Physical-Technical Institute, 194021 St. Petersburg, Russia

## S1. MOLECULAR ADSORPTION ENERGY AND SIMULATION OF SURFACE DIFFUSION

The diffusion coefficient of molecules on a surface strongly depends on temperature $\mathcal{T}$ and the activation energy for diffusion $E_{\mathrm{diff}}$ [1]. The latter is related to the adsorption energy $E_{\mathrm{ads}}$ for the molecule on the substrate, leading to the following relation:

$$D(\mathcal{T}, E_{\mathrm{ads}}) = D_0 \exp\left(-\frac{E_{\mathrm{ads}}}{k\mathcal{T}}\right), \tag{S1}$$

where $D_0$ is the diffusion coefficient at very large temperatures and $k$ is the Boltzmann's constant. The adsorption energy $E_{\mathrm{ads}}$ depends on the strength of the adhesion forces between the molecule and the substrate.

The molecule-substrate forces (arising in our simulations from van der Waals interactions between pairs of atoms) are modelled in molecular dynamics (MD) by means of the Lennard-Jones potential, which needs two parameters (interatomic distance and depth of the potential well). For W, C, Si, O and H atoms, different sets of van der Waals parameters can be found in the literature [2–5]. In the present study, these parameters have been combined in different ways, producing several sets which allow screening a wide region of potential adsorption energies, until reaching a diffusion coefficient in reasonable agreement with the only available (to the best of our knowledge) empirical information (estimated from the adjustment of model calculations to experimental data on nanostructure growth) [6]. Note that the choice could have been also performed relying on *ab initio* calculations of the adsorption energy, although we preferred to rely here on empirical information.

The diffusion coefficients and adsorption energies of $W(CO)_6$ molecules on hydroxylated $SiO_2$ have been obtained from MD simulations of 2 ns duration at $\mathcal{T} = 300$ K for each set of parameters, as explained in [2]. The results are depicted in Fig. S1 by symbols; the solid line represents the best fit by means of Eq. (S1). The experimentally determined value [6] is shown by a dashed line (the adsorption energy was not empirically obtained). An adsorption energy around 2.5 eV yields a surface diffusion coefficient of 7.71 $\mu m^2/s$, fairly close to the experimentally determined value of 6.64 $\mu m^2/s$ [6]. Parameters for C, H and O atoms come from [2], those for Si from [3], while for W they come from [5].

## S2. MONTE CARLO CODE SEED FOR ELECTRON TRANSPORT AND INTERACTION CROSS SECTIONS FOR SILICA

The details of the SEED (Secondary Electron Energy Deposition) Monte Carlo (MC) code for energetic electron transport in solids are explained in detail in [7]. It is based on the calculation of (i) the differential inelastic scattering cross sections obtained by using the dielectric formalism [8, 9], (ii) the electron-phonon quasi-elastic scattering cross-sections computed by the use of the Fröhlich theory [10] and (iii) the differential elastic scattering cross sections performed by the relativistic partial wave expansion method (RPWEM) [11] including the Ganachaud and Mokrani empirical correction for low electron energies ($\leq$ 20–30 eV) [12]. It should be noted that coherent scattering effects due to the long De Broglie wavelength for very low energy electrons are not yet considered in any MC code. Nonetheless, the good comparison of the results obtained by means of SEED with experimental and reference data (see below or [7] for further examples) demonstrates that its accuracy is good enough for the purposes of the present investigation.

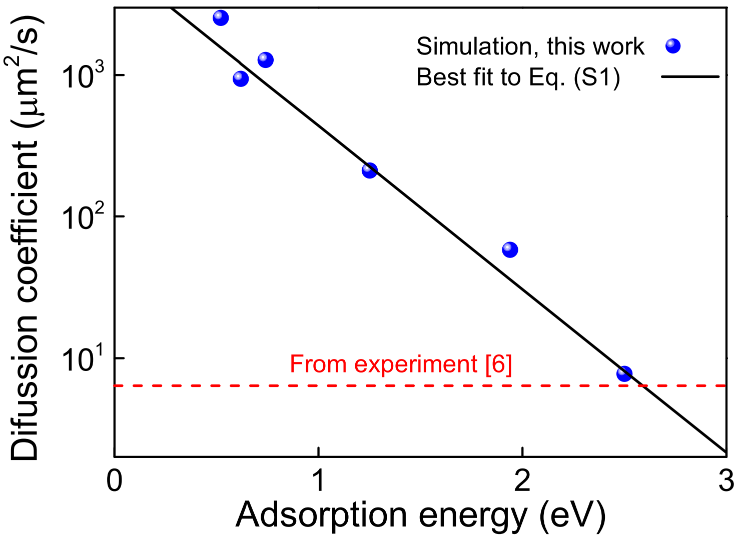


FIG. S1. **Determination of molecular adsorption energy and diffusion coefficient.** $W(CO)_6$ surface diffusion coefficient on hydroxylated $SiO_2$ as a function of the adsorption energy for different sets of van der Waals parameters. An empirically estimated value (the adsorption energy was not determined) is shown by a dashed line [6].

The dielectric formalism, very successful for the calculation of electronic interaction cross sections in condensed matter, requires the knowledge of the energy-loss function (ELF) of the target material, $\mathrm{Im}[-1/\epsilon(k,\omega)]$ (where $\epsilon(k,\omega)$ is the complex dielectric function), accounting for its electronic excitation spectrum for excitations of given energy and momentum, $\hbar\omega$ and $\hbar k$, respectively. An estimate for the mean binding energy of the outer-shell electrons of the target is needed in order to disentangle the processes of ionisation and electronic excitation [8]. The optical ELF ($\hbar k = 0$) of $SiO_2$ (mass density 2.19 g/cm$^3$) has been taken from the compilation of [13] and extended to finite momentum transfers by means of the Mermin Energy Loss Function-Generalised Oscillator Strengths (MELF-GOS) method [14], tested for many condensed-phase materials. A mean binding energy of 12.2 eV has been estimated from data taken from [15]. The effect of surface hydroxylation in the electronic properties of $SiO_2$ is not deemed to affect much the electron propagation through the substrate, which mostly occurs within the bulk of the material. The calculated total inelastic mean free path is shown by a solid line in Fig. S2(a) and compared to the available experimental data [16–19], finding a rather good agreement in a wide energy range.

The elastic mean free path for electrons in $SiO_2$ calculated by meas of the RPWEM is shown in Fig. S2(b) (dotted line). This method is known to provide unreasonable small values (shorter than interatomic distances) for $T < 10$ eV. To amend this tendency, the Ganachaud and Mokrani empirical correction is used [12], which requires the setting of the Ganachaud-Mokrani parameter $\alpha$. Figure S2(b) shows the effect of different choices of $\alpha$ in the resulting elastic mean free path. Furthermore, the Fröhlich theory [10] for electron-phonon scattering requires the knowledge of the characteristic phonon energy, $W_{\mathrm{ph}}$, whose typical values are in the range $0.01 - 0.1$ eV.

All elastic, inelastic and electron-phonon interactions have an impact in the trajectories of (especially low energy) electrons, affecting their ability to escape from the substrate and hence determining the secondary electron (SE) yield. Since inelastic and elastic (high energy)

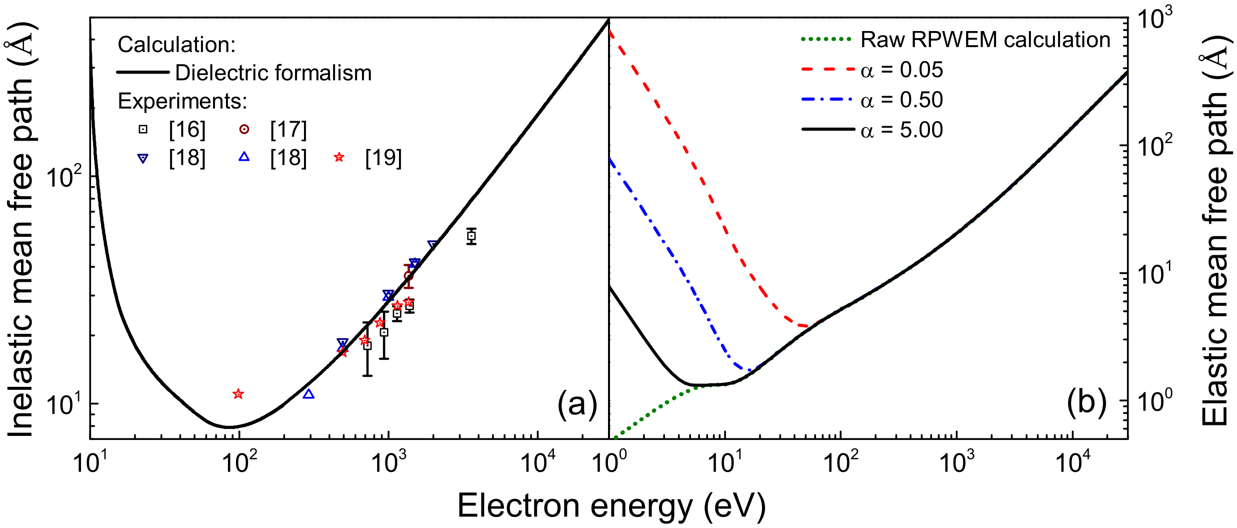


FIG. S2. **Mean free paths for electrons in silica.** (a) Calculated inelastic mean free path for electrons in $SiO_2$ (solid line) compared to experimental data (symbols) [16–19]. (b) Calculated elastic mean free path for electrons in $SiO_2$: the dotted line depicts the raw RPWEM calculation, while other lines show the results obtained with different choices of the Ganachaud-Mokrani parameter $\alpha$.

cross sections are fully determined (the former from the dielectric theory and the latter from the RPWEM calculations), only the setting of the free parameters for the Ganachaud-Mokrani and Fröhlich theories remains. These parameters have been set in order to correctly simulate the experimentally known SE yield for $SiO_2$ [20]. Figure S3(a) shows the effect of different choices of the Ganachaud-Mokrani parameter in the simulated SE yield (assuming $W_{\rm ph} = 0.15$ eV), while Fig. S3(b) shows the effect of different phonon energies (fixing $\alpha = 5$).

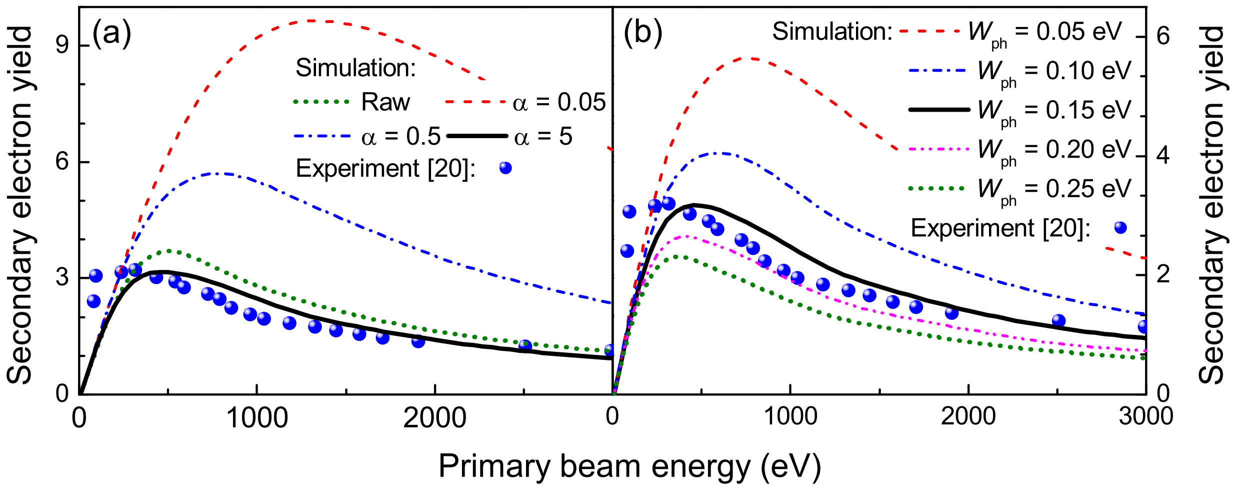


FIG. S3. **Secondary electron yield from silica.** Calculated secondary electron yield (a) for different values of the Ganachaud-Mokrani parameter $\alpha$ (lines), and (b) for different values of the phonon energy $W_{\rm ph}$, as compared to the experimental values (symbols) [20].

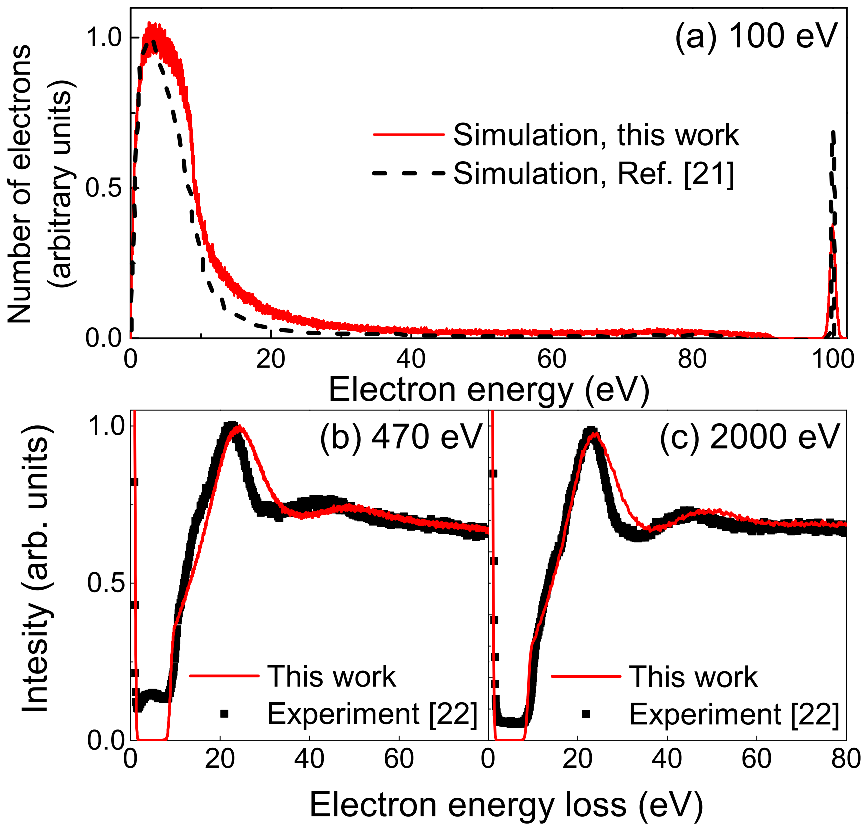


FIG. S4. **Energy loss of electrons in silica.** (a) Simulated SE and BSE energy spectrum for a 100 eV PE beam impinging in $SiO_2$: present calculation (solid histogram) and simulations from [21] (dashed histogram). The other panels depict simulated reflection electron energy loss spectra (REELS, lines) for PE beams of (b) 470 eV and (c) 2 keV, compared to experiments (symbols) [22].

Values of $\alpha = 5$ and $W_{\mathrm{ph}} = 0.15$ eV (solid lines) provide a very reasonable agreement with the experimental data (symbols) [20].

The reliability of the MC simulations has been tested by calculating the energy spectra as well as energy loss spectra of the reflected electrons (i.e., the reflection electron energy loss spectra, REELS) and comparing them to reference and experimental data. Figure S4(a) compares the calculated (solid histogram) energy spectrum of ejected SE and backscattered electrons (BSE) for a 100 eV primary electron (PE) beam impinging in $SiO_2$ with reference simulations (dashed histogram) [21]. Figures S4(b) and (c) compare the simulated REELS spectra (red lines) with experimental data (black symbols) [22] for PE beams of 470 eV and 2000 eV, respectively. Simulations qualitatively agree with the energy spectrum from [21] and are in an excellent agreement with the experimental REELS spectra [22].

## S3. $W(CO)_6$ MOLECULE FRAGMENTATION CROSS SECTIONS

For the $W(CO)_6$ molecule, experimental measurements exist for the *relative* cross sections for electron-impact dissociative ionisation (DI) [23] and for lower energy channels [24], although no information about the *absolute* cross sections is available.

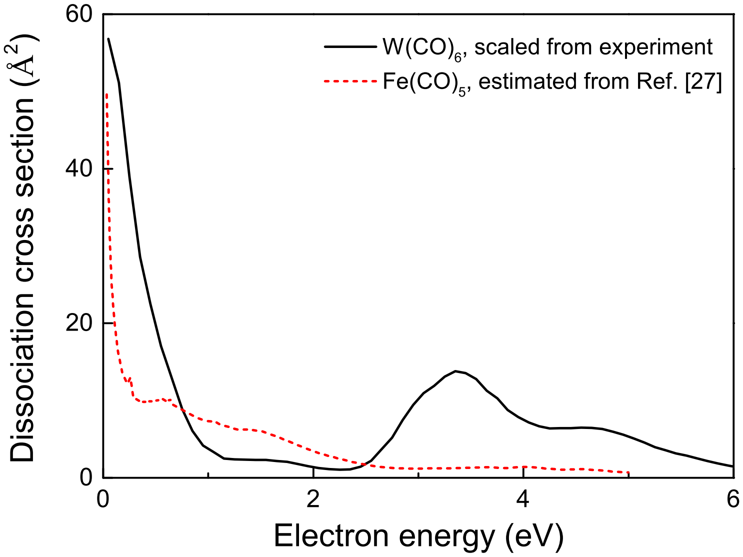


FIG. S5. **Electron-impact fragmentation cross section of W(CO)$_6$.** Comparison of the scaled low energy dissociation cross section for W(CO)$_6$ (this work) with that of Fe(CO)$_5$ (estimated from experiments and calculations from [27]).

The experimental data on DI relative cross sections reveals that almost every ionising collision ($\geq$ 97%) leads to W(CO)$_6$ fragmentation to some extent. Thus, it is possible to approximate the total DI cross section, $\sigma_{\mathrm{DI}}(T)$, by estimating the total ionisation cross section employing the dielectric formalism [8, 9] (see Supplementary Information S2). The optical ELF of W(CO)$_6$ has been estimated from a parametric approach for organic materials [8, 25] and a binding energy for the outer-shell electrons of 8.47 eV has been estimated from [23]. The calculated DI cross section is shown in Fig. 1(c) of the main text.

The absolute value of the lower energy dissociation channels cross section, $\sigma_{\mathrm{low}-T}(T)$, can be fixed by calculating the experimentally known decomposition cross section for W(CO)$_6$ molecules on SiO$_2$ by a PE beam of energy $T_0$:

$$\sigma_{\mathrm{decomp}}(T_0) = \frac{1}{N_{\mathrm{PE}}} \int_0^{T_0} \frac{\mathrm{d}N(T)}{\mathrm{d}T} \sigma_{\mathrm{frag}}(T)\mathrm{d}T \,, \qquad \text{(S2)}$$

where $\mathrm{d}N(T)/\mathrm{d}T$ is the energy spectrum of PE and the generated SE and BSE crossing the surface near the beam area (further normalised to the number $N_{\mathrm{PE}}$ of PE) and $\sigma_{\mathrm{frag}}(T) = \sigma_{\mathrm{DI}}(T) + \sigma_{\mathrm{low}-T}(T)$. The decomposition cross section has been experimentally determined to be 1.2 Å$^2$ for 30 keV PE [26]. The energy spectrum is obtained from MC simulations (see Fig. 1(b) of the main text). The experimental relative low energy dissociation cross section $\sigma_{\mathrm{low}-T}(T)$ [24] has been scaled so the integral of Eq. (S2) gives a value of 1.2 Å$^2$.

The resulting low energy cross section is shown by a solid line in Fig. S5, and is compared with that for the similar metal carbonyl Fe(CO)$_5$. For the latter molecule, measurements of the relative cross sections also exist, as well as calculations of its absolute values [27], which allow estimating the absolute cross section (dashed line in Fig. S5). The scaled cross section for W(CO)$_6$ is comparable with the absolute cross section for Fe(CO)$_5$, presenting the main characteristic peak (corresponding to the loss of a single CO ligand [24]), of a similar height, at $T \sim 0$ eV. Other dissociation channels observed for W(CO)$_6$ at 3.3 and

4.7 eV (corresponding to the loss of two and three CO ligands, respectively [24]) seem to be much weaker in $Fe(CO)_5$. The good comparison validates the scaling procedure employed.

## S4. IRRADIATION DRIVEN MOLECULAR DYNAMICS SIMULATIONS OF THE FEBID PROCESS WITH MBN EXPLORER

The fundamentals of irradiation driven molecular dynamics (IDMD) are summarised in "Methods" and full details can be found in [2]. Here, the main aspects influencing simulations within the current investigation are explained.

### A. Scaling of primary electron beam currents in simulations

The molecular fragmentation rate is influenced (see "Methods") by the PE flux, i.e., the number $N_{\mathrm{PE}}$ of delivered PE per unit time $t$ and unit area $S$ at the irradiated spot:

$$J_0 = \frac{I_0}{eS_0}\,, \tag{S3}$$

where $I_0 = e\,\mathrm{d}N_{\mathrm{PE}}/\mathrm{d}t$ is the PE beam current, $S_0 = \pi R^2$ is the PE beam area (with $R$ being the beam radius) and $e$ the elementary charge. Typical FEBID experiments use a beam radius of several nanometres. Here, it was set to $R = 5$ nm. For simplicity, uniform PE fluxes $J_0$ were simulated by the MC code SEED (Supplementary Information S2) within the beam area $S_0$. In all MBN Explorer simulations, the 20×20 nm$^2$ substrate is covered by 1–2 layers of $W(CO)_6$ molecules (density 5.4 molecules/nm$^2$), which guarantees the full coverage of the substrate while keeping a layer thin enough to not significantly affect PE trajectories.

Currents of the order $I_0 \sim$ pA–nA for irradiation (dwell) times $\tau_{\mathrm{d}} \sim \mu$s or longer are commonly used in experiments. However, currently it would be computationally very costly to perform MD simulations for such long times. Instead, in this work a number of PE per unit area and per dwell time similar to experiments was sought. For this purpose, the simulated currents (and hence the fluxes) are scaled in the following manner [2]:

$$\frac{I_0^{\mathrm{sim}}}{I_0^{\mathrm{exp}}} = \lambda \frac{S_0^{\mathrm{sim}}}{S_0^{\mathrm{exp}}} = \lambda \frac{R^{\mathrm{sim}\,2}}{R^{\mathrm{exp}\,2}}\,, \tag{S4}$$

where $\lambda = \tau_{\mathrm{d}}^{\mathrm{exp}}/\tau_{\mathrm{d}}^{\mathrm{sim}}$; the super-indexes "sim" refer to simulation beam parameters while "exp" to experimental beam parameters. In such a way, simulated beam currents $I_0^{\mathrm{sim}} \sim \mu$A give the same amount of PE per simulated dwell time $\tau_{\mathrm{d}}^{\mathrm{sim}} \sim$ ns (which is feasible for MD simulations) as experimental beam currents $I_0^{\mathrm{exp}} \sim$ nA for experimental dwell times $\tau_{\mathrm{d}}^{\mathrm{exp}} \sim \mu$s. In Table S1, the PE beam parameters corresponding to each simulation (this work) and each experiment from [28] (to which we compare) are summarised.

### B. Energy deposited to fragmenting bonds

Bond dissociation events are simulated by the deposition of an average energy per electron-molecule collision, $E_{\mathrm{dep}}$, in the atoms forming the bond, so that the atomic velocities increase fulfilling the requirements of momentum conservation [29]. For the sake of

TABLE S1. Beam parameters corresponding to each simulation (present work) and to each experiment reported in [28]. Simulations and experiments marked in bold were performed with similar PE fluxes.

| Simulation | $T_0$ (keV) | $R$ (nm) | $\tau_d$ (ns) | $I_0^{sim}$ ($\mu$A) | $I_0^{exp}$ (nA) | PE flux (elec./nm$^2$/$\tau_d$) |
|---|---|---|---|---|---|---|
| 0.5keV@0.25nA | 0.5 | 5 | 10 | 0.625 | 0.25 | 500 |
| 0.5keV@5.9nA | 0.5 | 5 | 10 | 14.75 | 5.9 | 11700 |
| **1keV@3.7nA** | **1** | **5** | **10** | **9.25** | **3.7** | **7352** |
| **10keV@2.3nA** | **10** | **5** | **10** | **5.75** | **2.3** | **4570** |
| **30keV@0.28nA** | **30** | **5** | **10** | **0.7** | **0.28** | **556** |
| 30keV@5.9nA | 30 | 5 | 10 | 14.75 | 5.9 | 11700 |
| Experiment | $T_0$ (keV) | $R$ (nm) | $\tau_d$ ($\mu$s) | $I_0^{sim}$ ($\mu$A) | $I_0^{exp}$ (nA) | PE flux (elec./nm$^2$/$\tau_d$) |
| 4keV@6.6nA | 4 | 10 | 5 | - | 6.6 | 656 |
| 5keV@3.2nA | 5 | 10 | 100 | - | 3.2 | 6371 |
| 5keV@3.3nA | 5 | 10 | 100 | - | 3.3 | 6589 |
| **5keV@3.7nA** | **5** | **10** | **100** | **-** | **3.7** | **7352** |
| 5keV@4.1nA | 5 | 10 | 100 | - | 4.1 | 8189 |
| **11keV@2.3nA** | **11** | **10** | **100** | **-** | **2.3** | **4570** |
| 17keV@1.52nA | 17 | 10 | 100 | - | 1.52 | 3020 |
| **20keV@0.25nA** | **20** | **10** | **100** | **-** | **0.25** | **497** |
| 20keV@0.5nA | 20 | 10 | 100 | - | 0.5 | 994 |
| 20keV@0.51nA | 20 | 10 | 100 | - | 0.51 | 1013 |
| **24keV@0.28nA** | **24** | **10** | **100** | **-** | **0.28** | **556** |

simplicity, in this work it is assumed that every fragmentation event leads to the cleavage of a single W-C bond, while the much stronger C-O bonds will stay intact [29]. Further collisions of the fragments with the environment may lead to subsequent cleavage of additional W-C bonds [29].

The choice of $E_{dep}$ influences the kinetics of the electron-driven chemical reactions, since low values favour metal-ligand recombination after dissociation, while larger values effectively put the metal atom and the ligand apart. A value of $E_{dep} = 325$ kcal/mol ($\sim 14$ eV) has been chosen in simulations, since it is consistent with average values obtained from mass spectrometry experiments [24, 30] and dedicated $W(CO)_6$ fragmentation simulations [29].

Unfortunately, the kinetics of $W(CO)_6$ molecules fragmentation has not been measured on $SiO_2$ substrates, although it is known for gold substrates [31]. In [31], 1–2 layers of $W(CO)_6$ molecules were deposited on Au, as in present simulations in $SiO_2$, and the effective decomposition cross section $\sigma_{decomp}(T_0)$ (see Supplementary Information S3) was measured for a $T_0 = 500$ eV broad PE beam. On the one hand, this dense packing of molecules on the substrate prevents surface diffusion to some extent. On the other hand, the broad macroscopic beam (of $\sim 1$ cm$^2$ area [31]) characterised by a decomposition cross section $\sigma_{decomp}(T_0)$ produces a uniform fragmentation probability $P = J_0\, \sigma_{decomp}(T_0)$ over a nanometric area. As a consequence, it might be possible to, at least qualitatively, compare simulations on $SiO_2$ to the reported experiments on a Au substrate [31], despite their potential differences in terms

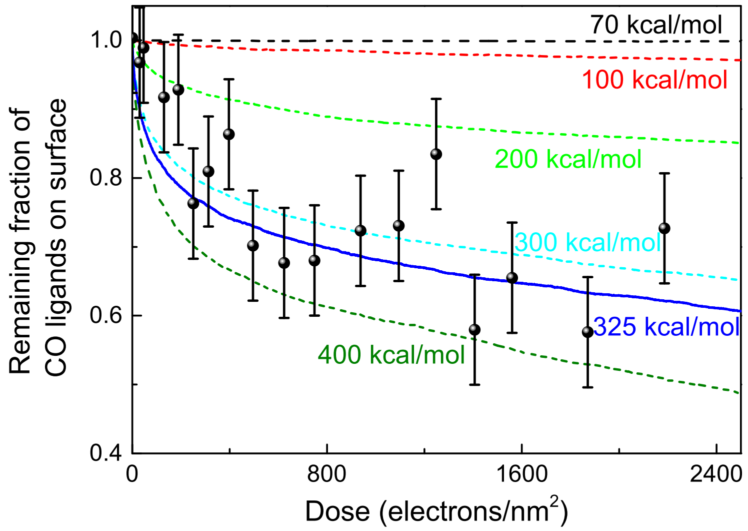


FIG. S6. **Electron-induced degradation of $W(CO)_6$ molecules.** Fraction of CO ligands remaining on a gold substrate covered with $W(CO)_6$ molecules as a function of the PE dose delivered by a uniform 500 eV beam. Symbols represent experimental data [31], while lines are simulation results using the indicated average deposited energies per collision.

of surface diffusion and electron emission, provided that the empirical decomposition cross section is employed for these specific simulations.

The measured fraction of CO ligands remaining on the Au substrate as a function of the PE dose is shown by symbols in Fig. S6. IDMD simulations have been performed for an spatially uniform beam, employing the experimentally determined decomposition cross section $\sigma_{\rm decomp}(T_0)$ for $W(CO)_6$ molecules on gold [31]. Note that, in this particular case, there is no need to rely on MC simulation results, since the fragmentation rate $P = J_0\,\sigma_{\rm decomp}(T_0)$ is constant all over the simulated surface. The value $E_{\rm dep}$ has been scanned in the range 70–400 kcal/mol, consistent with gas-phase findings [24, 30]. As can be seen from the simulated curves, a value of 300–325 kcal/mol ($\sim$13–14 eV) reproduces fairly well the main features of the experimental data, this value reasonably agreeing with the average deposited energies estimated from gas-phase mass spectrometry [24, 30]. This comparison further supports our choice for $E_{\rm dep}$.

### C. Models for the chemistry of reactive sites within the growing nanostructures

Once molecules are fragmented and chemically reactive sites start to appear in the system, atoms with dangling bonds will start to form new bonds as they encounter other atoms with unsaturated valencies, following the rules of the reactive force field [32]. In a previous work [2], two models for the formation of new chemical bonds were introduced: model A only accounts for the chemistry between newly added precursors and the growing deposit, without restructuring of the dangling bonds within the latter (i.e., the search for reactive neighbours is done only among the atoms located beyond the molecular structure to which a chosen atom belongs). Model B allows for the formation of new bonds within the deposit itself (i.e., the search is performed over all atoms with dangling bonds in the simulation box, including the molecular structure to which a chosen atom belongs). As can be seen in the main text,

consideration of these two different chemistry models A and B can lead to slightly different deposit compositions. However, the variations observed in simulations are similar to, or even lower than, the deviations observed in experimental measurements [28], so the accuracy of the present simulations is comparable to that of available experimental techniques.

---


[1] Utke, I., Hoffmann, P. & Melngailis, J. Gas-assisted focused electron beam and ion beam processing and fabrication. *Journal of Vacuum Science & Technology B: Microelectronics and Nanometer Structures* **26**, 1197–1276 (2008).

[2] Sushko, G. B., Solov'yov, I. A. & Solov'yov, A. V. Molecular dynamics for irradiation driven chemistry: application to the FEBID process. *The European Physical Journal D* **70**, 217 (2016). URL `http://link.springer.com/10.1140/epjd/e2016-70283-5`.

[3] Athanasopoulos, D. C. & Garofalini, S. H. Molecular dynamics simulations of the effect of adsorption on $SiO_2$ surfaces. *The Journal of Chemical Physics* **97**, 3775–3780 (1992).

[4] Bernardes, C. E., Canongia Lopes, J. N. & Da Piedade, M. E. All-atom force field for molecular dynamics simulations on organotransition metal solids and liquids. Application to $M(CO)_n$ (M = Cr, Fe, Ni, Mo, Ru, or W) compounds. *Journal of Physical Chemistry A* **117**, 11107–11113 (2013).

[5] Filippova, V. P., Kunavin, S. A. & Pugachev, M. S. Calculation of the parameters of the Lennard-Jones potential for pairs of identical atoms based on the properties of solid substances. *Inorganic Materials: Applied Research* **6**, 1–4 (2015).

[6] Fowlkes, J. D. & Rack, P. D. Fundamental Electron-Precursor-Solid Deposition Simulations and Experiments. *ACS Nano* **4**, 1619–1629 (2010). URL `http://www.ncbi.nlm.nih.gov/pubmed/20201541`.

[7] Dapor, M. *Transport of Energetic Electrons in Solids. Computer Simulation with Applications to Materials Analysis and Characterization* (Springer International Publishing AG, Cham, Switzerland, 2020), 3 edn.

[8] de Vera, P., Garcia-Molina, R., Abril, I. & Solov'yov, A. V. Semiempirical Model for the Ion Impact Ionization of Complex Biological Media. *Physical Review Letters* **110**, 148104 (2013).

[9] de Vera, P. & Garcia-Molina, R. Electron inelastic mean free paths in condensed matter down to a few electronvolts. *Journal of Physical Chemistry C* **123**, 2075–2083 (2019). URL `http://arxiv.org/abs/1807.11710`. 1807.11710.

[10] Fröhlich, H. Electrons in Lattice Fields. *Advances in Physics* **3**, 325–361 (1954).

[11] Jablonski, A., Salvat, F. & Powell, C. J. Comparison of Electron Elastic-Scattering Cross Sections Calculated from Two Commonly Used Atomic Potentials. *Journal of Physical and Chemical Reference Data* **33**, 409–451 (2004). URL `http://link.aip.org/link/?JPR/33/409/1&Agg=doi`.

[12] Ganachaud, J. P. & Mokrani, A. Theoretical study of the secondary electron emission of insulating targets. *Surface Science* **334**, 329–341 (1995).

[13] Palik, E. D. & Ghosh, G. *The Electronic Handbook of Optical Constants of Solids* (Academic Press, San Diego, 1999).

[14] Garcia-Molina, R., Abril, I., Heredia-Avalos, S., Kyriakou, I. & Emfietzoglou, D. A combined molecular dynamics and Monte Carlo simulation of the spatial distribution of energy deposition by proton beams in liquid water. *Physics in Medicine and Biology* **56**, 6475–6493 (2011). URL `http://stacks.iop.org/0031-9155/56/i=19/a=019?key=crossref.`

f03df0d5516820cb8f88b4c438553668.

[15] Garvie, L. A., Rez, P., Alvarez, J. R. & Buseck, P. R. Interband transitions of crystalline and amorphous $SiO_2$: An electron energy-loss spectroscopy (EELS) study of the low-loss region. *Solid State Communications* **106**, 303–307 (1998).

[16] Flitsch, R. & Raider, S. I. Electron mean escape depths from x-ray photoelectron spectra of thermally oxidized silicon dioxide films on silicon. *Journal of Vacuum Science and Technology* **12**, 305–308 (1975). URL http://link.aip.org/link/?JVS/12/305/1%5Cnhttp://scitation.aip.org/content/avs/journal/jvst/12/1/10.1116/1.568771.

[17] Hill, J. M., Royce, D. G., Fadley, C. S., Wagner, L. F. & Grunthaner, F. J. Properties of oxidized silicon as determined by angular-dependent X-ray photoelectron spectroscopy. *Chemical Physics Letters* **44**, 225–231 (1976).

[18] Jung, R. *et al.* Determination of effective electron inelastic mean free paths in $SiO_2$ and $Si_3N_4$ using a Si reference. *Surface Science* **543**, 153–161 (2003).

[19] Murat, M., Akkerman, A. & Barak, J. Spatial distribution of electron-hole pairs induced by electrons and protons in $SiO_2$. *IEEE Transactions on Nuclear Science* **51**, 3211–3218 (2004).

[20] Glavatskikh, I. A., Kortov, V. S. & Fitting, H. J. Self-consistent electrical charging of insulating layers and metal-insulator-semiconductor structures. *Journal of Applied Physics* **89**, 440–448 (2001).

[21] Schreiber, E. & Fitting, H.-J. Monte Carlo simulation of secondary electron emission from the insulator $SiO_2$. *Journal of Electron Spectroscopy and Related Phenomena* **124**, 25–37 (2002).

[22] Filippi, M., Calliari, L. & Dapor, M. Joint experimental and computational study of silicon dioxide electron energy loss spectra. *Physical Review B* **75**, 1–6 (2007). URL http://link.aps.org/doi/10.1103/PhysRevB.75.125406.

[23] Wnorowski, K., Stano, M., Barszczewska, W., Jówko, A. & Matejčík, S. Electron ionization of $W(CO)_6$: Appearance energies. *International Journal of Mass Spectrometry* **314**, 42–48 (2012).

[24] Wnorowski, K. *et al.* Low-energy electron interactions with tungsten hexacarbonyl - $W(CO)_6$. *Rapid Communications in Mass Spectrometry* **26**, 2093–2098 (2012).

[25] Tan, Z. *et al.* Electron stopping power and mean free path in organic compounds over the energy range of 20–10,000 eV. *Nuclear Instruments and Methods in Physics Research Section B: Beam Interactions with Materials and Atoms* **222**, 27–43 (2004). URL http://linkinghub.elsevier.com/retrieve/pii/S0168583X04003283.

[26] Hoyle, P. C., Cleaver, J. R. A. & Ahmed, H. Ultralow-energy focused electron beam induced deposition. *Applied Physics Letters* **64**, 1448–1450 (1994).

[27] Allan, M. *et al.* Dissociative electron attachment and electronic excitation in $Fe(CO)_5$. *Physical Chemistry Chemical Physics* **20**, 11692–11701 (2018).

[28] Porrati, F., Sachser, R. & Huth, M. The transient electrical conductivity of W-based electron-beam-induced deposits during growth, irradiation and exposure to air. *Nanotechnology* **20**, 195301 (2009).

[29] de Vera, P., Verkhovtsev, A., Sushko, G. & Solov'yov, A. V. Reactive molecular dynamics simulations of organometallic compound $W(CO)_6$ fragmentation. *European Physical Journal D* **73**, 215 (2019).

[30] Cooks, R. G., Ast, T., Kralj, B., Kramer, V. & Žigon, D. Internal energy distributions deposited in doubly and singly charged tungsten hexacarbonyl ions generated by charge stripping, electron impact, and charge exchange. *Journal of the American Society for Mass Spectrometry* **1**, 16–27 (1990).

[31] Rosenberg, S. G., Barclay, M. & Fairbrother, D. H. Electron induced reactions of surface adsorbed tungsten hexacarbonyl ($W(CO)_6$). *Physical Chemistry Chemical Physics* **15**, 4002–4015 (2013). URL http://xlink.rsc.org/?DOI=c3cp43902j.

[32] Sushko, G. B., Solov'yov, I. A., Verkhovtsev, A. V., Volkov, S. N. & Solov'yov, A. V. Studying chemical reactions in biological systems with MBN Explorer: implementation of molecular mechanics with dynamical topology. *European Physical Journal D* **70**, 12 (2016). URL http://arxiv.org/abs/1507.03423. 1507.03423.
